# Potential protective effects of chrysin against immunotoxicity induced by diazinon

**DOI:** 10.1038/s41598-022-20010-3

**Published:** 2022-09-16

**Authors:** Majid Zeinali, Azam Shafaei, Houshang Rafatpanah, Arman Mosavat, Naser Tayebi-Meybodi, Hossein Hosseinzadeh, Seyed Abdolrahim Rezaee

**Affiliations:** 1 Pharmaceutical Research Center, Pharmaceutical Technology Institute, School of Pharmacy, Mashhad University of Medical Sciences, Azadi-Square, Medical Campus, Mashhad, 9177948954 Iran; 2 Social Security Organization (SSO), Mashhad, Razavi Khorasan Iran; 3 Blood Borne Infections Research Center, Academic Center for Education, Culture, and Research (ACECR), Mashhad, Razavi Khorasan Iran; 4 Immunology Research Center, Inflammation and Inflammatory Diseases Division, Faculty of Medicine, Mashhad University of Medical Sciences, Azadi-Square, Medical Campus, Mashhad, 9177948564 Iran; 5 Department of Pathology, Faculty of Medicine, Mashhad University of Medical Sciences, Mashhad, Iran; 6 Department of Pharmacodynamics and Toxicology, School of Pharmacy, Mashhad University of Medical Sciences, Mashhad, Iran

**Keywords:** Cytokines, Gene regulation in immune cells, Inflammation, Lymphocytes, Immunology

## Abstract

Acute intoxication with diazinon (DZN) as a pesticide causes mortality and morbidity annually. This study shows the impact of sub-acute toxicity of DZN 20 mg/kg and the protective activities of chrysin (CH) as a flavone under the flavonoids family (12.5, 25 and 50 mg/kg) were assessed on BALB/c mouse immune system. The changes in morphological and functional properties of the immune system on thymus, spleen and liver histopathology, sub-populations of T lymphocytes, cytokines levels, transcription factors, complement function, phagocytosis, specific and total antibody productions were considered. The histopathological effects of DZN on the spleen and thymus were not significant, but the liver was damaged remarkably. In the presence of CH, the toxic effect of DZN is suppressed. DZN significantly decreased the number of whole blood TCD4^+^, TCD8^+^ and NK cells and suppressed the phagocytosis, delayed-type hypersensitivity (DTH) responses to sheep red blood cell (SRBC). Furthermore, it suppressed specific anti-SRBC-Ab, total IgG and IgM production, T-bet expression, and IFN-γ production. In contrast, DZN did not significantly affect complement function and the number of NK cells, TCD4^+^ and TCD8^+^ splenocytes. However, it potentiated the expression of *GATA-3*, *ROR-γt* and *FOXP3* gene expression and consequently produced IL-4, IL-10, IL-17 and TGF-β in whole blood. CH not only significantly increased the variables mentioned above at 12.5, 25 and 50 mg/kg but also could overcome the toxic effects of DZN on whole blood lymphocyte sub-populations and specific and total Ab production in 25 and 50 mg/kg concentrations, phagocytosis and DTH responses in 50 mg/kg, and modulation of the transcription factors and cytokine production, mainly in 25 and 50 mg/kg. In conclusion, DZN in sub-acute doses could remarkably deteriorate immune responses. However, CH can overcome the toxic effects of DZN on the immune components and functions of the immune system.

## Introduction

Organophosphorus pesticides (OPs) are a group of insecticides derived from phosphoric or phosphorothioate acid^1^. In vivo and in vitro studies demonstrated that exposure to these pesticides exhibits several toxic effects^2^. Acute intoxication with OPs is estimated to cause more than three million life-threatening poisonings each year, over 250,000 deaths of self-poisoning and about one-third of the world's suicide cases^3^.

Diazinon (DZN), one of the organophosphate insecticides, has been widely used in agriculture in recent years. Several studies in rabbits, rats and mice have shown that DZN can induce biochemical, physiological, and histopathological alterations in many organs such as the liver, kidney, heart, testis, and brain^4–6^.

Organophosphorus pesticides, including DZN, are cholinesterase inhibitors that inhibit acetylcholinesterase (AChE) activity. The primary function of AChE is to terminate nerve impulses through the hydrolysis of the neurotransmitter (Ach). Accordingly, AChE is a complex component of the cholinergic activities in brain synapses and neuromuscular junctions^7^. However, some observations indicate that not the full effects of OPs insecticides are due to AChE inhibition^8^. The mechanism underlying the effects of acute OPs intoxication is not well understood, but inflammation and oxidative stress are supposed to be critical intermediates in chronic exposure, notably^3^. DZN has been reported to increase tumour necrosis factor-alpha (TNF-α) production in rat serum and brain. Although, the study findings in low doses of DZN on immune systems have been challenging. DZN in low doses can cause toxic effects on animal blood cells, spleen, thymus, and lymph nodes, lymphocyte and macrophage functions suggesting that it has destructive effects on the immune system^9–12^.

According to the oxidative and pro-inflammatory effects of DZN on the immune system, flavonoids as antioxidant and anti-inflammatory agents might effectively neutralise the harmful effects of such toxins^13^.

Flavonoids belong to a group of plant-origin compounds with variable phenolic structures. Over the last two decades, flavonoids have gained significant attention in treating disorders with their biochemical and pharmacological properties, such as pro-oxidant and antioxidant potential that correlate to their structure–activity relationships^14^. Antioxidants exert various radical scavenging properties by inhibiting chelated trace elements or enzymes that generate excessive reactive oxygen species (ROS) and/or by enhancing endogenous antioxidant enzymes such as glutathione peroxidase (GPx), catalase (CAT), and superoxide dismutase (SOD). Flavonoids regulate transcription factors such as transcription factor nuclear factor-κB (NF-κB), nuclear factor erythroid 2-related factor 2 (Nrf2), and Activator protein 1 (AP-1) in inflammation, DNA damage, and cell cycle^15^.

Chrysin (CH) (5,7-dihydroxyflavone) as a flavonoid can be found in various herbal plants, particularly in *Passiflora incarnata*^16^. Recently, several studies have shown that selected flavonoids, for example, CH, have multiple putative in vitro and in vivo biological activities, such as anti-inflammatory, antioxidant, anti-atherogenic, and anti-diabetic^13,17,18^. Flavonoids have been suggested as therapeutic herbal agents in inflammatory reactions triggered by neutrophilFurthermore; they have immunomodulatory properties via various activities of the immune system cells such as THs, CTLs, B cells, and NK cells^13,17,18^. Moreover, CH has been known as the antagonist of NF-κB and the agonist of peroxisome proliferator-activated receptor-gamma (PPAR-γ), which can down-regulate the key pro-inflammatory enzymes such as myeloperoxidase (MPO), cyclooxygenase-2 (COX-2), inducible nitric oxide synthase (iNOS), phospholipase A2, and prostanoids^13^. It could also inhibit the levels of major inflammatory cytokines, the activity of iNOS, and consequently, plasma NO levels in rats^19^.

In the present study, the potential immunoprotective effects of CH, a *Passiflora incarnata* extract, were evaluated in DZN-induced sub-acute toxicity on lymphoid organs such as the spleen, and thymus and liver in mice. Furthermore, the impact of DZN and CH were analytically assessed on the T cell subpopulation in terms of phenotypes, transcription factors and cytokines production. Finally, the effector phases of immune responses, specific Ab production and delayed hypersensitivities responses were also investigated to comprehensively understand the toxic effects of DZN and the anti-toxic properties of CH on the immune system.

## Results

### Histopathological findings

The histopathological evaluation of CH and DZN on the liver, kidney, and heart was demonstrated previously^6^. Thus, the DZN impact in the presence or absence of CH was explained in this section on the main lymphoid organs. The thymus, liver and spleen were studied as primary and secondary lymphoid organs. As the liver is also a secondary lymphoid organ, this study showed the immunological changes findings.

### Thymus

**Figure 1:**
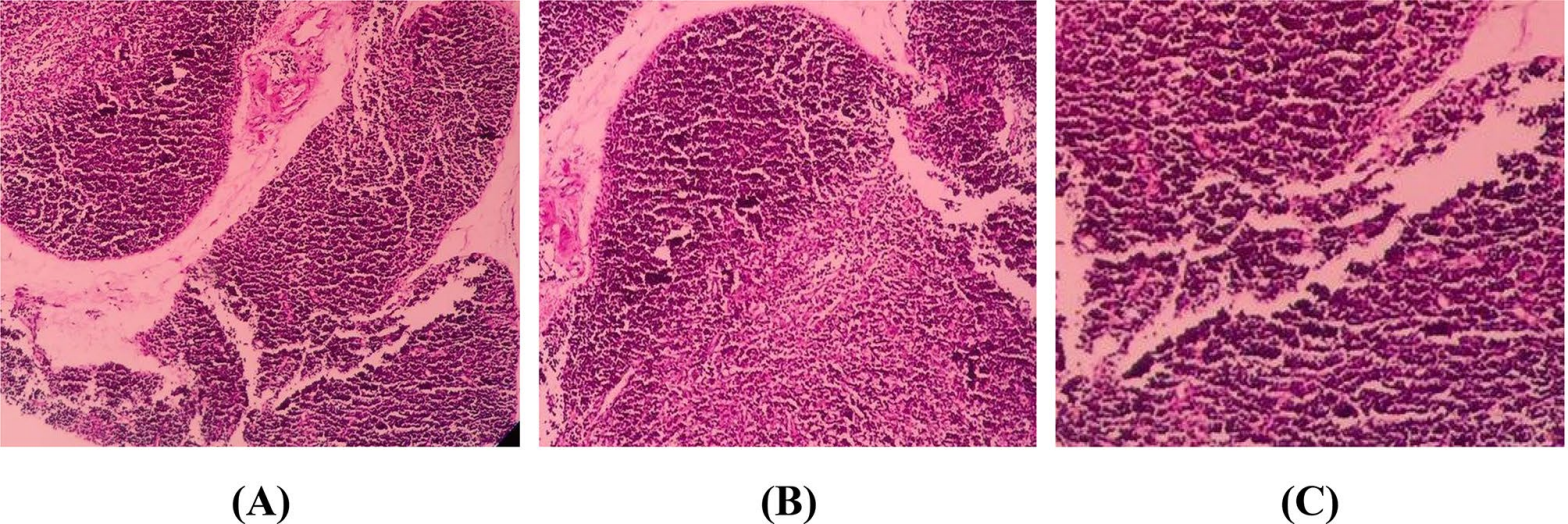
The histopathological effects of CH and DZN on the BALB/c thymus in studied groups. The mice thymus section in control group (**A**), DZN (20 mg/kg) group (**B**), and DZN plus CH 50 mg/kg (**C**). The pathological examination did not show any main difference in the presence or the absence of CH. The cytopathology of the cortex and medulla showed no inflammatory reactions or necrosis. Hematoxylin and Eosin (H&E) staining, × 400 magnification.

The pathological examination of the thymus in the DZN group did not show any significant difference in the presence or the absence of CH 50 mg/kg. The cytopathology of the cortex and medulla did not show significant inflammatory reactions or necrosis in the studied groups (Fig. 1).

### Liver

**Figure 2:**
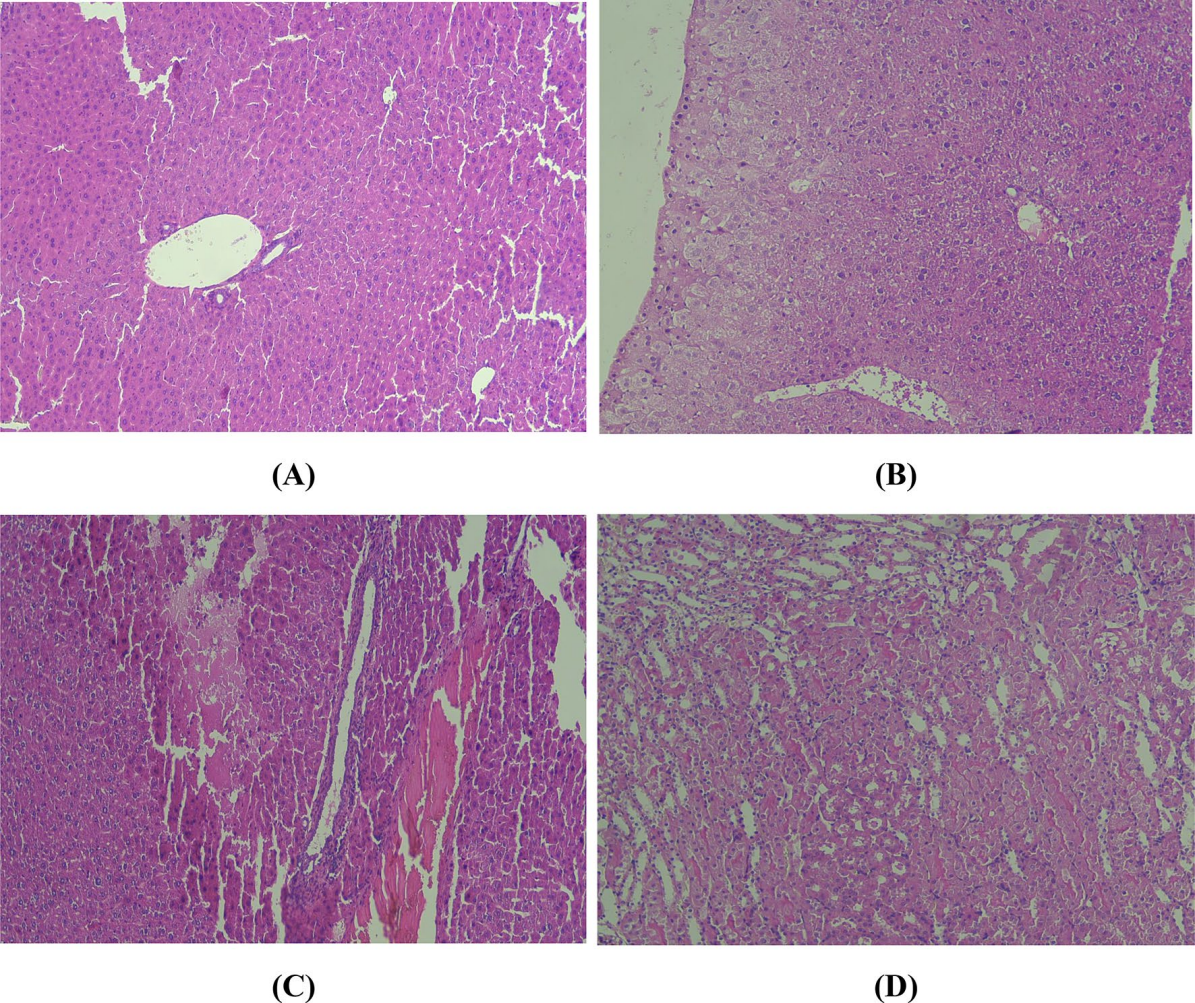
The histopathological effects of CH and DZN on the BALB/c liver in studied groups. The mice liver section in the control group (**A**). As the microscopic findings showed, DZN induced focal necrosis in an acute inflammation with infiltration of granulocytes and mononuclear cells in liver tissue (**B**). Furthermore, the clostasis was observed in the liver section (**C**). While, whereas CH in the highest concentration (50 mg/kg) was able to nearly reduce the toxic effects of DZN in the DZN plus CH group (**D**). Hematoxylin and Eosin (H&E) staining, × 400 magnification.

The toxic effects of DZN in histopathologic experiments were very serious. However, the CH could ameliorate this toxicity in DZN plus CH group (Fig. 2). As the microscopic findings showed, DZN induced an acute inflammation along with focal necrosis with infiltration of granulocytes and mononuclear cells in liver tissue (Fig. 2B). Furthermore, the clostasis also could be observed in the liver (Fig. 2C). On the other hand, CH in the highest concentration (50 mg/kg) was able to nearly reduce the toxic effects of DZN in DZN plus CH group (Fig. 2D).

### Spleen

**Figure 3:**
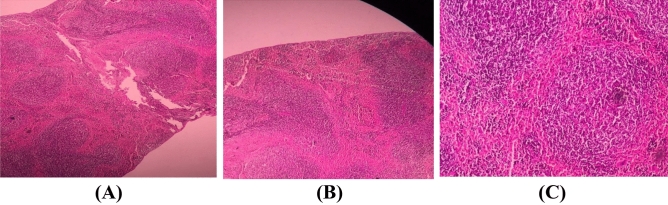
The histological effect of CH and DZN on the BALB/c spleen changes induced by DZN in studied groups. The mice spleen section in control group (**A**), DZN (20 mg/kg) group (**B**), and DZN plus CH 50 mg/kg (**C**). The impact of CH on the spleen in DZN treated and untreated mice showed significant extra-medullary hematopoietic congestion. There were no significant histopathologic changes among the studied groups. Hematoxylin and Eosin (H&E) staining, × 400 magnification.

The impact of CH 50 mg/kg on the spleen in the DZN group comparing the control group showed significant extra-medullary hematopoietic congestion. On the other hand, no significant effect has been observed among the studied group samples regarding the extra-medullary hematopoietic rate (Fig. 3).

### Splenocyte subtypes

The flowcytometry test with specific conjugated antibodies for evaluation of the spleen lymphocyte subpopulations illustrated that DZN could cause a significant reduction in the percentage and number of TCD4^+^, TCD8^+^, and NK cells. However, in the presence of CH, even though it could somehow neutralise the effect of DZN and increase the percentage and the number of those cells, the data analysis showed no significant differences. Table 1 shows the effects of DZN and CH on splenocyte subpopulations.

**Table 1:** The results of DZN 20 mg/kg and CH 50 mg/kg, and DZN plus CH (12.5, 25, and 50 mg/kg) on splenocyte subtypes in BALB/c mice.

	DZN	DZN + CH1	DZN + CH2	DZN + CH3	CH3	CTX	Control
Spleen Cell Numbers (× 10^7^)	8.38 ± 0.03*	8.61 ± 0.16	9.37 ± 0.25	9.61 ± 0.15 +	10.43 ± 0.58	8.08 ± 0.04**	9.54 ± 0.11
CD19^+^ Cell (%)	32.20 ± 3.08	34.80 ± 1.31	35.4 ± 0.6	37.61 ± 0.81	39.60 ± 0.5	18.60 ± 1.32***	37.60 ± 3.18
CD19^+^ content	2.53 ± 0.26*	2.99 ± 0.12	3.31 ± 0.07	3.61 ± 0.11 + +	4.12 ± 0.23	1.50 ± 0.10***	3.59 ± 0.32
CD49b^+^ cell (%)	3.94 ± 0.53***	4.26 ± 0.55	4.87 ± 0.48	6.07 ± 0.4 +	6.88 ± 0.23	3.90 ± 0.24***	7.72 ± 0.44
CD49b^+^ content (× 10^7^)	0.33 ± 0.04***	0.36 ± 0.04	0.45 ± 0.04	0.58 ± 0.01 + + +	0.71 ± 0.03	0.31 ± 0.2***	0.73 ± 0.04
CD3^+^ cell (%)	48.2 ± 1.59***	53.8 ± 1.95	58.4 ± 0.92 + +	57.4 ± 2.73 + +	72.6 ± 1.32	43.4 ± 1.2***	70.4 ± 1.69
CD3^+^ content	3.37 ± 0.34	5.12 ± 0.32	5.44 ± 0.17	5.09 ± 0.19	6.16 ± 0.32	5.80 ± 0.21***	5.62 ± 0.10
CD4^+^ cell (%)	40.20 ± 4.09*	45.4 ± 1.36	44 ± 1.51	45.6 ± 1.63	44.8 ± 1.24	39.8 ± 0.58*	49.20 ± 1.06
CD4^+^ content	3.37 ± 0.34**	3.91 ± 0.17	4.12 ± 0.18	4.38 ± 0.16 +	4.67 ± 0.29	3.21 ± 0.06***	4.69 ± 0.14
CD8^+^ cell (%)	20.04 ± 0.79*	20.42 ± 0.47	21.01 ± 0.41	22.64 ± 0.26	25.04 ± 0.59	16.34 ± 1.11***	24.37 ± 1.87
CD8^+^ content	1.68 ± 0.06**	1.75 ± 0.03	1.97 ± 0.05	2.17 ± 0.05 +	2.61 ± 0.18	1.32 ± 0.09***	2.32 ± 0.14

Data showed as mean ± SD, (*) showed significant effect compared to control, ( +) comparison with the diazinon-treated group. *p* < 0.05 and (***) or (+ +  + +) *p* < 0.001. DZN caused a significant reduction in the percentage and number of TCD4^+^, TCD8^+^, and NK cells. However, in the presence of CH, it could somehow neutralise the effect of DZN and increase the percentage and the number of immune cells.

### Phagocytosis capacity

According to the peripheral blood flow cytometry results, the statistical analyses showed that DZN at a dose of 20 mg/kg body weight reduced phagocytic activities compared to the control group. CH at 50 mg/kg increased phagocytic activity compared to the control group. CH only at a dose of 50 mg/kg in the presence of DZN also neutralised the suppression activities of DZN on phagocytosis. However, CH at the lowest concentration (12.5 mg/kg) could not change the impact of DZN on phagocytic activity (Table 2).

**Table 2:** Effect of DZN 20 mg/kg and CH 50 mg/kg, and DZN plus CH (12.5, 25, and 50 mg/kg) on phagocytic capacity of neutrophils and monocytes in BALB/c mice.

	DZN	DZN + CH1	DZN + CH2	DZN + CH3	CH3	X	Control
Fluorescence intensity/phag ocytic cell	15.30 ± 0.46***	15.21 ± 0.3	17 ± 0.48 +	20.12 ± 0.1 + + +	24.90 ± 0.3***	12.64 ± 0.2***	20.04 ± 0.6

Data showed as mean ± SD, (*) shows the significant changes compared to controls, ( +) comparison with DZN-treated group. *p* < 0.05 and (***) or (+ +  + +) *p* < 0.001. DZN at a dose of 20 mg/kg significantly reduced phagocytic activity compared to the control group. CH, in a dose-dependent manner, significantly increased phagocytic activity compared to the control group.

### The delayed-type hypersensitivity test (DTH)

The statistical analyses revealed that DZN reduced the DTH reactions in SRBC sensitised animals compared to the control group after 24 h. CH at doses of 12.5, 25, and 50 mg/kg strongly neutralised the effect of DZN after 24 h. In contrast, CH only at the highest dose of 50 mg/kg was able to neutralise the impact of DZN after 48 h on DTH responses in animals receiving DZN plus CH (Table 3).

**Table 3:** Effect of DZN 20 mg/kg and CH 50 mg/kg, and DZN plus CH (12.5, 25, and 50 mg/kg) on DTH responses in BALB/c mice.

Groups	DTH 24 h	DTH 48 h
Control	26.37 ± 0.13	13.33 ± 1.99
DZN	14.54 ± 0.05 ***	8.83 ± 0.7
DZN + CH1	16.25 ± 0.01 + + +	9.66 ± 2.45
DZN + CH2	18.64 ± 0.16 + + +	9.67 ± 1.72
DZN + CH3	19.42 ± 0.19 + +	17.50 ± 2.21 +
CH3	23.01 ± 0.03 ***	18.83 ± 1.19
CTX 20 mg/kg	12.62 ± 0.06 ***	5.33 ± 0.55 *

Data showed as mean ± SD, (*) shows the significant changes compared to controls, ( +) comparison with the diazinon-treated group. *p* < 0.05 and (***) or (+ +  + +) *p* < 0.001. DZN significantly reduced the DTH in sensitised animals compared to the control group after 24 h. CH at doses of 12.5, 25, and 50 mg/kg strongly neutralised the effect of DZN after 24 h. However, CH only at the highest dose of 50 mg/kg was able to neutralise the impact of DZN after 48 h on DTH responses in animals receiving DZN plus CH.

### Humoral immunity

The haemagglutination test (HA) results revealed that in the DZN group, the production of specific anti-SRBC for evaluation of specific humoral immunity decreased. However, CH at the highest concentrations, 25 and 50 mg/kg, was able to neutralise the inhibitory effect of DZN on specific antibody production. As an immunoinhibitory drug, dexamethasone also dramatically suppressed the specific antibody production compared to the control group. Table 4 shows the results in different groups.

**Table 4:** The results of DZN 20 mg/kg, CH 50 mg/kg, and CH and DZN plus CH (12.5, 25, and 50 mg/kg) on cytokine and total IgM and IgG production in BALB/c mice.

Parameters	DZN	DZN + CH1	DZN + CH2	DZN + CH3	CH3	CTX	Control
IL-4 (pg/mL)	26.23 ± 0.34***	25.87 ± 0.82	24.91 ± 1.14	22.76 ± 0.51 + +	22.52 ± 0.27	11.63 ± 0.28***	21.67 ± 0.41
IL-10 (pg/mL)	32.47 ± 0.50***	30.66 ± 1.08	31.88 ± 0.32	29.41 ± 0.49 +	24.44 ± 0.36	12.11 ± 0.72***	26.36 ± 0.56
IFN-γ (pg/mL)	16.55 ± 0.36***	17.93 ± 0.45	18.46 ± 0.53	20.62 ± 0.76 + + +	22.98 ± 0.49	14.24 ± 0.50***	24.29 ± 0.66
TGF-β (pg/mL)	34.67 ± 0.89***	31.02 ± 0.59 + +	29.33 ± 0.82 + + +	28.18 ± 0.83 + + +	24.24 ± 0.22	14.52 ± 0.69	26.17 ± 0.68
IL-17 (pg/mL)	19.27 ± 0.15***	19.07 ± 0.13	18.80 ± 0.30	17.14 ± 0.21 + +	14.15 ± 0.26	11.72 ± 0.71***	13.94 ± 0.21
IgM (μl/ml)	1022.4 ± 7.19***	1044 ± 15.03	1087 ± 2.7 + +	1103 ± 4.77 + + +	1154 ± 9.27	879 ± 7.48***	1106 ± 23.12
IgG (μl/mL)	8236 ± 23.99***	8304 ± 32.96	8595 ± 13.23 + + +	9009 ± 20.46 + + +	9823 ± 13.18 ***	7524 ± 20.91 ***	8541 ± 24.29

Data showed as mean ± SD, (*) shows the significant changes compared to controls ( +) comparison with DZN-treated group. *p* < 0.05 and (***) or (+ +  + +) *p* < 0.001. Cytokines measurement in the DZN group showed that the production of IFN-γ in the DZN group was significantly down-regulated compared to the control group. However, the significant impact of CH on the neutralisation of toxicity effects of DZN on IFN-γ was dose-dependent and only taken at the highest concentration of CH 50 mg/kg.

Furthermore, ELISA tests showed that total IgM and IgG in toxin-receiving animals significantly decreased. At the same time, CH at doses of 25 and 50 mg/kg had a strong ability to increase the amount of IgG and neutralise the effect of DZN (Table 4).

Moreover, complement activity also decreased in the DZN-treated mice, but the differences among studied groups were not statistically significant (Table 5).

**Table 5:** The results of DZN 20 mg/kg and CH 50 mg/kg, CH and DZN plus CH (12.5, 25, and 50 mg/kg) on CH50 and specific Ab production in BALB/c mice.

Groups	CH50 (u/µL)	Anti-SRBC, HA titre (log 2)
Control	45.67 ± 2.14	7.6 ± 0.24
DZN	43.5 ± 1.87	6.2 ± 0.73 **
DZN + CH_1_	46.33 ± 2.53	7 ± 031
DZN + CH_2_	50 ± 2.30	7.2 ± 0.37 +
DZN + CH_3_	48 ± 2.26	7.8 ± 0.2 + +
CH3	49 ± 1.88	7.8 ± 0.37
Dexamethasone 4 mg/kg		6.2 ± 0.73 **

Data showed as mean ± SD, (*) shows the significant changes compared to controls, ( +) comparison with the diazinon-treated group. *p* < 0.05 and (***) or (+ +  + +) *p* < 0.001. The results of the HA test showed that producing specific anti-SRBC for evaluation of humoral immunity inhibited when animals received DZN. However, CH at the highest concentration, 50 mg/kg, was able to neutralise the inhibitory effect of DZN strongly. Albite and CH at doses of 12.5 and 25 mg/kg also statistically ameliorated the toxic effects of DZN plus CH. As an immune-inhibitory drug, dexamethasone also dramatically suppressed the specific antibody production compared to the control group. Moreover, complement hemolytic activity decreased in the DZN-receiving animal, but the result was not statistically significant.

### Cytokine genes expression assay

The RT-qPCR, EvaGreen® method was used in the study of *RORγt*, *GATA3*, *T-bet*, *FOXP3*, *IL-4*, *IL-10*, *IFN-γ*, *TGF-β*, and *IL-17* expression, as transcription factors and cytokine in Th subpopulations in peripheral blood (Th1, Th2, Th17 and Treg). Table 6 shows the increased expression of *IL-4*, *IL-10*, *IL-17*, *TGF-β*, *GATA3*, and *RORγt* genes in the DZN group compared to the control group. CH ameliorated the effects of DZN in treated mice with CH plus DZN.

**Table 6:** The results of DZN 20 mg/kg, CH 50 mg/kg and CH and DZN plus CH (12.5, 25, and 50 mg/kg) on cytokine gene expression in BALB/c mice.

Target genes	DZN	DZN + CH1	DZN + CH2	DZN + CH3	CH3	CTX	Control
*IL-4*	125.49 ± 19.16**	57.87 ± 11.8 + + +	63.58 ± 3.09 + + +	52.81 ± 6.81 + + +	69.19 ± 8.49	26.85 ± 4.50***	73.52 ± 4.50
*IL-10*	58.70 ± 6.19***	32.30 ± 2.34 + + +	24.06 ± 0.33 + + +	19.70 ± 1.63 + + +	31.68 ± 0.92	13.71 ± 2.18***	34.84 ± 1.53
*IFN-γ*	127.08 ± 8.85***	166.48 ± 9.01	231.81 ± 17.85 + +	286.64 ± 20.5 + + +	326.78 ± 33.30	101.95 ± 8.31***	401.25 ± 18.04
*TGF-β*	52.51 ± 1.89***	36.65 ± 3.11 + + +	31.61 ± 2.10 + + +	32.72 ± 2.85 + + +	22.67 ± 1.57	12.15 ± 0.93***	23.94 ± 0.89
*IL-17*	1780 ± 184.94***	998.21 ± 8.97 + +	827 ± 41.58 + + +	797 ± 25.38 + + +	735.46 ± 14.29	403.77 ± 31.64***	847.11 ± 3.48
*RORγ*	849.52 ± 52.42***	645.25 ± 36.49 + +	640.36 ± 10.47 + + +	536.51 ± 51.26 + + +	662.33 ± 11.45	382.01 ± 39.98***	620.98 ± 12.58
*GATA3*	934.92 ± 50.61***	785.21 ± 66.99	604.59 ± 50.53 + +	442.83 ± 67.83 + + +	378.58 ± 24.31	204.94 ± 58.73***	355.56 ± 15.69
*T-bet*	4701.9 ± 898.8***	4829.2 ± 435.6	8333.7 ± 539 + +	9721.8 ± 517.9 + + +	10,840 ± 907.4	4263.8 ± 584.6***	8641.96 ± 161.2
*FOXP3*	55.78 ± 2.43***	89.49 ± 7.65	172.38 ± 11.72 + + +	191.77 ± 11.05 + + +	300.28 ± 20.34	27.22 ± 4.60***	260.955 ± 8.05

Data showed as mean ± SD, (*) shows the significant changes compared to controls, ( +) comparison with DZN-treated group. *p* < 0.05 and (***) or (+ +  + +) *p* < 0.001. RT-qPCR, EvaGreen method was used to study the gene expression as transcription factors and cytokine for Th subpopulations (Th1, Th2, Th17, Treg). Data showed the increased expression of *IL-4*, *IL-10*, *IL-17*, *TGF-β*, *GATA3*, and *RORγt* genes in the DZN treated group compared to the control group. However, CH was able to up-regulate the toxic effect of DZN. On the other hand, DZN significantly down-regulated the expression of *IFN-γ*, *FOXP3* and *T-bet* genes. However, CH could substantially modulate the decrement effect of DZN on these factors at different doses.

On the other hand, DZN significantly down-regulated the expression of *IFN-γ*, *FOXP3* and *T-bet* genes. In the presence of CH, the decrement effect of DZN on these factors at different doses was improved (Table 6).

### Cytokine production

Cytokines measurement in supernatants of splenocytes in the DZN group (Table 4) disclosed that the production of IFN-γ in the DZN group was significantly down-regulated compared to the control group (16.55 ± 0.36 vs 24.29 ± 0.66, respectively, *p* = 0.001).

Nevertheless, the effect of CH for neutralising the toxic effects of DZN on IFN-γ was dose-dependent and only effective at the highest concentration of CH 50 mg/kg (*p* = 0.001), i.e*.,* the production of this cytokine in DZN plus CH in 12.5, 25 and 50 mg/kg were 17.93 ± 0.45, 18.46 ± 0.53 and 20.62 ± 0.76, respectively.

Consistent with the selective transcription factors for inducing cytokine production, the findings disclosed that in the DZN group, the expression of *IL-17, IL-10, IL-4,* and *TGF-β* were up-regulated compared to the control group.

## Discussion

Organophosphorus pesticides are used in agriculture extensively. Widespread use of Ops results in contamination of air, water, soil, farmers and agricultural resources^20^. These pesticides inhibit acetylcholinesterase (AChE), the critical enzyme involved in nervous system functions. However, OPs have many other toxic effects, including high genotoxicity, hepatic dysfunction, embryotoxicity, and induction of neuro-behavioural and neurochemical changes^21^. On the other hand, herbal medicine is most common among farmers to prevent such toxicities. In the present study, the impacts of DZN on the immune system components were evaluated, and this opinion was examined on whether CH as a potent flavonoid has protective effects on the toxic properties of DZN.

In general, the results of our previous study defined that DZN caused changes in cell blood counts and histological changes in tissues of BALB/c mice. This study demonstrated that CH at higher doses strongly reduced the toxic effects of DZN^6^.

The most harmful mechanisms of Ops, such as DZN, our intervention in the oxidative stress and cholinergic signalling pathways and, consequently, the manifestation of the diseases^10,22^. Organophosphates such as DZN increase the molecular metabolites of oxidative stress inside the cells of vital organs such as the heart, liver, and lungs and cause severe chronic damage^23^.

Like other studies, we have shown that chrysin as a flavonoid reduces the pathological effects of DZN on tissues, especially the liver, as a pivotal metabolic and immune system organ^6,24^. The hepato-protective activity of CH has been associated with improving antioxidant defence and potentiates the production of SOD, CAT, GPx activity and GSH levels^25^. Moreover, it has been suggested that CH inhibits hepatic oxidative stress through regulation of the extracellular signal-regulated kinase (ERK)2/nuclear factor-erythroid-2-related factor 2 (Nrf2)/antioxidant response element (ARE) pathway^26^. The present histopathological tissue analysis showed no significant complications in other lymphoid organs such as the spleen and thymus in the DZN group. This is a challenging result compared to other studies which reported toxic effects of DZN on the spleen and thymus. In such studies, the level and treated duration with DZN have been higher and longer^27,28^.

In the present study, the concentration of DZN has been chosen near the frequent doses a farmer may receive. Therefore, these methodological differences can considerably diverge the studies' results. Although, DZN did not have significant histopathologic changes in the spleen and thymus. The cellular finding identified that the main subpopulations of the immune cells in the spleen and peripheral blood had been affected by DZN. For example, flowcytometry of splenocyte suspension showed that DZN caused a significant reduction in the number of phenotypic markers, including CD4, CD8, CD19, and CD49. These markers represent special populations/or subpopulations of immune cells. CD19 represents the B cell population which forms humoral immunity. CD4 molecule represents the Th lymphocyte subpopulation, the central cell of the immune responses in the presence of any danger signals. CD8 represents the Ag-specific cytolytic lymphocytes, the main component of anti-cancer and anti-intracellular infectious agents such as viruses, bacteria, and fungi^29–31^.

Consequently, by reducing such lymphocyte subpopulations, DZN leads to severe or chronic damage to the immune responses. Such damages have been reported in farmers exposed to DZN^32^. While flavonoids, in our focus, chrysin can neutralise the harmful effects of pesticides and toxic medications^33^.

Reactive oxygen species have been associated with T lymphocyte unresponsive and apoptosis. Furthermore, ROS by the decrement production of signalling factors such as ERK, PLC-γ, AKT in activation and T-bet and STAT-1 in differentiation to the effector T cells. These signalling pathway alterations lowered the production of cytokines, such as IL-4, IL-2 and IFN-γ^34^. Thus, in the presence of a high concentration of ROS during inflammatory reactions, ROS are harmful by-products for the appropriate functions of T lymphocytes as the central immune cells helping all arms of defence^34–36^. Such conditions may cause T cell response alterations and lead to the development of chronic diseases or cancers^37^. Establishing a balance between oxidant and antioxidant defence production is essential for proper cellular activity in the immune system^38,39^. On the other hand, CH can modulate the innate immune system by inhibiting inflammatory reactions.

This study demonstrated that CH could overcome the changes caused by DZN on B cells, Th and CTLs. Thus, CH not only increases the number of these immune cells but also potentiates their activities. Furthermore, a significant increase in the total and relative weight of the spleen in the CH-or DZN plus CH groups confirmed that this change might be due to increment of cell content such as T cell, B cell, and NK cell populations in the presence of CH. Likewise, in animals receiving DZN plus CH, the number of natural killer cells in the spleen strongly increased, which was another reason CH overcame the toxic impact of DZN and strengthened the immune system^40^. Together, these findings demonstrated that CH strongly suppressed inflammatory reactions in the innate immune system. It seems that these properties of CH might be due to modulation of the main inflammatory cells, i.e*.* neutrophils and macrophages^41–43^.

Phagocytosis activities of neutrophils and monocyte/macrophages represent the power of their microbial killing. The present study showed the ingested FITC-labeled *E. coli* and the average number of ingested per cell has potentiated in the presence of CH, particularly at 50 mg/kg. In contrast, DZN at a dose of 20 mg/kg significantly reduced phagocytic activity compared to the control group. Besides, CH overcame the toxic effect of DZN at 50 mg/kg and increased phagocyte activity of phagocytic cells. However, 12.5 mg/kg of CH did not remarkably affect neutralising the DZN toxicity on phagocytic activity. The flavonoids are bioactive factors in the modulation of innate inflammatory cells, neutrophils and monocytes/macrophages; They suppress the inflammatory mediators of these cells, such as TNF-α, IL-1β, preformed and newly-formed mediators such as leukotrienes, prostaglandins, but also up-regulate the phagocytosis^2,44–46^.

It has been reported that in the presence of antioxidants, the phagocytic ability of macrophages increased^47,48^. Antioxidant impact on phagocytosis has been reported in various studies^13,49^. It can be concluded that CH strengthens the innate immune system in terms of phagocytic activities^17^. It has been suggested that the immuno-modulation activities of chrysin accomplish via regulation of peroxisome proliferator-activated receptor γ (PPARγ) function. This effective modulation of PPAR by chrysin prevents the damage of phagocytes' inflammatory reactions by inducing the M1/M2 balance of macrophages to M2 anti-inflammatory phenotype^50^. Therefore, in this respect, the therapeutic properties of chrysin are noticeable in autoimmunity, DTH-associated diseases, and intracellular infections by modulating M1/M2 status^50,51^.

The complement system plays a central role in the homeostasis and installing the innate and adaptive immune responses^52^. Our results clarified that the activity of the complement system has not been significantly changed in DZN or DZN plus CH groups.

Humoral immunity neutralises the microbes and their products by Abs and facilitates antigen removal by phagocytic cells^53^. Our study showed that DZN decreased the specific anti-SRBCs antibodies, but CH at 50 mg/kg increased the production of this specific Abs and total mouse IgM and IgG levels. Taken together, potentiation of Th and B cell responses in the presence of CH up-regulates B and T lymphocytes number and activities. Additionally, cellular immune response (CMI) to the SRBCs increased by CH because DTH responses potentiated as evidence of Th1 polarisation. This condition requires specific recognition of an antigen by activated T cells. In the present study, CH not only potentiated the DTH responses but also overcame the toxic effect of DZN. Albite, in this study, the potentiation of the IFN-γ secretion in a dose-dependent manner from splenocytes in the CH-treated group confirmed that the DTH reaction up-regulated. Other reports also demonstrated such results for CH, which induced the immune system toward Th1 immunity^51^.

The present study evaluated the selective transcription factors that induce the activities of Th subpopulations, such as Th1, Th2, Th17 and Treg, to produce proper cytokines. The activation of *the* T-bet transcription factor results in Th1 lymphocyte differentiation to produce IFN-γ and form CMI responses. GATA-3 activation induces the differentiation of Th0 toward Th2, which produces IL-4, helping B lymphocytes produce Ab. *ROR-γt* is the transcription factor for differentiation of Th17, producing IL-17 and activating inflammatory reactions. IL-10 and TGF-β cytokines represent T regulatory cells (CD4^+^, CD25^+^, FOX3^+^) as anti-inflammatory cells that modulate immune responses^54^.

In this study, analysis of cytokines in splenocyte supernatants in the DZN group significantly lowered the amount of IFN-γ. However, CH overcame the toxic effects of DZN at the highest dose (50 mg/kg). On the other hand, IL-17, IL-10, IL-4, and TGF-β increased in the DZN group, while the concentration of these cytokines decreased in the DZN-CH group treated with 50 mg/kg. Overall, suppression of IFN-γ reduced IgG in the DZN group and neutralised the toxic effects of DZN by CH, indicating a protective effect of this flavonoid on cellular and humoral immune responses.

DZN causes the most changes in Th1 epigenetic processes, leading to suppression of the CMI system, DTH reactions, specific anti-SRBCs and total IgG production. It seems that diazinon can interfere with cytokine production by affecting the epigenetic changes in the polarisation of T cell subpopulations. These compounds can harm the response in humoral and cellular immune systems by disrupting appropriate lymphocyte polarisation and effectively changing the fate of viral, bacterial, parasitic, allergic and cancerous diseases^50^.

Studies emphasised that DZN suppressed the production of IFN-γ and potentiated the secretion of IL-17, IL-10, IL-4, and TGF-β. These effects caused inappropriate changes in the Th1/Th2 and Th17/Treg balances, predisposing animals to infectious disease and cancers^55,56^. Up-regulation of *IL-4* and *TGF-β* expression in the DZN group, which changed in the presence of CH, can be explained from two different views. Firstly, increasing IL-4 can suppress CMI and changes the balance of Th1/Th2 toward Th2 responses in favour of increasing allergic diseases and susceptibility to intracellular microbes and cancer. Secondly, the increasing amount of TGF-β may cause an immunocompromised condition in which the DZN-exposed subjects become more vulnerable to microbial and cancer diseases. In contrast, CH can overcome these side effects and modulate the immune system toward appropriate immune responses.

The RT-qPCR, TaqMan method has been used to evaluate the transcriptional factors inducing epigenetics changes in Th toward its subpopulations^57–59^. The present study showed an increase in the expression of *GATA3* and *RORγt* transcription levels in the DZN-receiving group, which in turn, CH can neutralise those toxic effects. These results are in line with Th epigenetic changes in cytokine assay.

DZN also inhibited the expression of *T-bet* and *FOXP3* transcription factors significantly. However, CH at different doses modulated the expression of these transcription factors. Studies have shown that DZN up-regulated IL-4 and down-regulated IFN-γ production. As a result, the IL-4/IFN-γ ratio increased, which could alter the immune system balance, but CH can modulate this severe imbalance to appropriate conditions^60–62^. By reducing the expression of *FOXP3*, DZN can change the balance of Th17/Treg in favour of Th17 inflammatory cytokine. Similar effects of the Paraquat pesticide have also been observed, which induces such inflammatory reactions^63^.

There were some inconsistencies in the results of DZN on cytokine gene expressions (RT-qPCR assay) and production (ELISA method) among the studied groups (Tables 4 and 6). The findings must be interpreted as the impact of DZN on the stability of mRNA in gene expression experiments. It has been reported that DZN indirectly causes DNA and RNA damage by inducing ROS production; in particular, it suppresses mRNA translation of IL-2 and IFN-γ^55^.

In overall, CH could ameliorate the adverse effects of DZN in mice. Due to its anti-inflammatory properties, it seems a decreased expression of *TNF-α* and *NF-KB/P65*-mRNA, stimulation of cytokine secretion, potentiation of IL-4 and IL-10, and modulation of IFN-γ secretion from splenocytes and peripheral lymphocytes. Moreover, it affects monocyte and macrophage IFN-γ-dependent release and has several roles in reducing the toxic impact on the immune system^9^. Furthermore, CH had many beneficial inhibitory agents on the suppression of inflammatory signalling pathways such as NF-κB, p65 unit, TNF-α, IL-1β, IL-6, IL-12, IL-17A, and IFN-γ was reported. Moreover, CH has been known as the antagonist of NF-kB and the agonist of PPAR-γ, which down-regulation of the key pro-inflammatory enzymes such as MPO, COX-2, iNOS, phospholipase A2, and prostanoids. Therefore, CH can improve the immune system^13^.

## Conclusions

In conclusion, this study cleared that excessive use of pesticides, particularly DZN, causes several toxic effects on different arms of the immune system. Apart from the neurotoxic effects of DZN, gradual exposure to any organism, particularly humans, reduced the antioxidant capacity and deteriorated the signalling pathways of immune cells toward inappropriate immune responses. The immune system components are more sensitive to this pesticide, from decrement in some lymphoid and myeloid cells to down-regulation of immune cell activities and deterioration of appropriate immune responses. In the present study, CH as a potent antioxidant flavonoid could overcome the harmful effects of this pesticide in mice and improve the numbers and activities of Th lymphocytes, monocytes and B cells. Therefore, these activities of CH as an exogenous antioxidant shed light on the potential therapeutic properties as an immunomodulatory medication in immunopathologic diseases and cancers.

## Methods

### Experiments protocol

The Biomedical Research Ethics committee of Mashhad University of Medical Sciences (MUMS), Mashad, Iran, was reviewed and approved the animal experiments [IR.MUMS.REC.922238]. These study protocols were performed according to the Explanation and Elaboration for the ARRIVE guidelines 2.0. All methods were performed following relevant guidelines and regulations.

Male BALB/c mice, free-pathogen6-week-old, were purchased from the animal house of Pasture Institute of Iran (Tehran, Iran). Male BALB/c mice (21 ± 3 gr weight) were kept in standard conditions of light, temperature and feeding in Animal House, the School of pharmacology, Mashhad University of Medical Sciences, Mashad, Iran.

Mice were divided into seven groups: (I) DZN-administrated mice (Sigma–Aldrich, USA) (20 mg/kg/day, gavage)^6,64^; (II-IV) treated with different doses of CH (Sigma–Aldrich, USA) (12.5, 25, or 50 mg/kg/day, intraperitoneally) plus DZN (20 mg/kg/day, gavage)^6^; (V) treated with CH (50 mg/kg/day, i.p); (VI) control (corn oil, gavage) and (VII) treated with cyclophosphamide (Baxter, Germany) as positive control group. The animal treatment schedule was performed between 16:00 and 18:00 for 28 days.

After every step of the study, animals were euthanised, and the liver, thymus and spleen were excised aseptically for histopathological examination. The samples were routinely paraffin-embedded, and sections of 4–6 µm were prepared for processing. The tissue sections, after processing, were stained with hematoxylin–eosin (Merck, Germany). A pathologist subsequently examined histopathological evaluation in a double-blinded manner.

### Delayed-type hypersensitivity (DTH) assay

To assess Ag-specific DTH response, sheep red blood cells (RBCs) were used to experiment according to the Fararjeh et al. study^65^. After the mouse footpad challenging, the thickness was determined at 24 and 48 h by a calliper.

### Specific Ab and total immunoglobulin production measurements

Mice were intraperitoneally (IP) injected with 5 × 10^8^ RBCs on days 0, 7th and 14th. Two weeks after the last booster injection, venous blood was collected, and serum was separated. The serum sample was serially diluted (two-fold), dispensed into a 96-well V shape microtiter plate, and then mixed with SRBC for haemagglutination assay. The plate was incubated for 60 min at 37 °C. The highest dilution of a given sample with definite agglutination is the positive titer of anti-SRBC, a standard test for assessing specific immune responses to a specific target.

To measure total IgG and IgM in mice groups, ELISA kits were used (Thermo Fisher Scientific; Cat. No. 39-50,400 and 39-50,470, USA) according to the manufacturer's instructions.

### Complement activity (CH50) assay

For measuring the total complement activity (CH50) in serum, sensitised anti-SRBCs-IgG and RBCs were used to measure the CH50, in which the mice sera act as the source of the complement. Complement lysed the Ag-Ab complex and then determined the percentage of hemolysis relative to the 100% lysis control.

### Evaluation of splenocyte subpopulations

After euthanasia, the mouse spleen was removed aseptically, and splenocytes were prepared. Harvested splenocytes were then processed for the experiments, as previously described in detail ^66^. The number of cells was calibrated at 1 × 10^6^ cells/mL in each vial, and cells were incubated with anti-CD3ePE-CY7, anti-CD4PE, anti-CD8 FITC, anti-CD19 FITC, and anti-CD49b FITC (BD Pharmingen, USA) as control isotopes. The flowcytometry was carried out by the FACSCalibur™ system (BD, USA) according to the manufacturer's instructions.

### Cytokine assay

The harvested splenocytes from each animal were cultured in Hut medium and kept in an incubator at 5% CO_2_ and 95% humidity for 72 h. After incubation, the supernatants were collected and centrifuged at 10,000 g for 2 min at 4 °C. Finally, the supernatant was collected for cytokine evaluation in microtubes and stored at −70 °C. Cytokines production (IL-4, IL-10, IL-17, IFN-γ, and TGF-β) were assessed by commercial ELISA kits (eBioscience, Cat. No. BMS613, 88-7105-22, 88-8711-22, BMS606 and BMS608, USA), according to the manufacturer's instructions.

### Phagocytosis evaluation

Phagocytosis was assessed using the commercial kit PHAGOTEST™ Kit (Orpegen Pharma, USA) on WBC according to the manufacturer's instructions by a FACS Calibur™ system. The kit has Fluorescein-labeled opsonised *E. coli* as a foreign agent. The labelled *E.coli* was used to quantify the capacity of phagocytosis. Phagocytic potency was expressed as fluorescence intensity in each phagocytic cell.

### RNA isolation and cDNA synthesis

The RNA extraction was performed by the Tripura reagent according to the manufacturer's instruction (Roche, Cat. No. 11667165001, Germany). Then, the extracted RNA was reverse-transcribed to cDNA with RevertAid™ First Strand cDNA Synthesis Kit (Thermo Fisher Scientific, Cat. No. K1622, USA).

### Gene expression assessments using quantitative real-time PCR (RT-qPCR)

Primers for mouse *IL-4, IL-10, IL-17, FOXP3, IFN-γ, GATA3, RORγ, T-bet,* and *TGF-β* were designed by Beacon Designer software V. 7 (PREMIER biosoft, USA). The RT-qPCR, EvaGreen method was used for gene expressions assay. Primer sequences are shown in Table 7.

**Table 7:** Primer sequences were used in the study.

Target genes	Forward sequence (5'$\to$ 3')	Reverse sequence (5'$\to$ 3')
*GATA3*	GTTCGGATGTAAGTCGAG	CAGGCATTGCAAAGGTAG
*FOXP3*	GGCACTATCACACATAGG	GTGTCTGACTTGTATTTTGG
*RORγ*	CTGAGGCCATTCAGTATG	CTGCACATTCTGACTAGG
*T-bet*	GGAGGCTATTTATTGTAGAGA	CAGCAAACTTTGATCCAC
*IL-4*	GTCCTCACAGCAACGAAG	GCAGCTCCATGAGAACAC
*IL-10*	AATAAGAGCAAGGCAGTG	TCCAGCAGACTCAATACA
*IL-17A*	GCTGACCCCTAAGAAACC	GTGGAGGGCAGACAATTC
*IFN-γ*	GAATGTGTCAGGTAGTAA	AATGAGCGAGTTATTTGT
*TGF-β*	CGAAGCGGACTACTATGC	CTTCCCGAATGTCTGACG
*β-actin*	TAGGCGGACTGTTACTGAGC	TGCTCCAACCAACTGCTGTC

The reference sequence of genes was obtained from the National Center for Biotechnology Information (NCBI), and then the complementary bindings of the primers were checked by nucleotide and PCR blast databasehttps://www.ncbi.nlm.nih.gov/tools/primer-blast/.

The method of RT-qPCR was a relative two-standard curves method using EvaGreen (Qiagen, Germany) by a Rotor-Gene *Q*-6000 machine (Qiagen, Germany). Beta-actin was used as a reference gene, six-point standard curves were created, and data was analysed using Rotor-Gene *Q* software (Qiagen, Germany). The copies of mRNA for each sample were obtained using this equation: copies number of the gene of interest/copies number of reference gene and optimised to present the gene expression index.

### Statistical analysis

Data were analysed by the SPSS software ver.11.5 (SPSS, Chicago, IL). Descriptive analyses were carried out using mean ± SD. For analytic calculations, one-way ANOVA with Tukey post-test and student *t*-test was used to compare the quantitative variables among and between studies groups, respectively. The differences were considered statistically significant if the *p*-value ≤ 0.05.

### Ethical approval

The animal experiments were reviewed and approved, by the Institutional Animal Care and Use Committee of Mashhad University of Medical Sciences [IR.MUMS.REC.922238]. During the experiments, the mice were monitored every day. All surgery was performed under sodium pentobarbital anaesthesia, and all efforts were made to minimise suffering.

## Data Availability

All datasets generated or analysed during the current study are included in this paper and are available from the corresponding author upon reasonable request.

## Abbreviations

CAT: Catalase
CH: Chrysin
CH50: Complement activity
DTH: Delayed-type hypersensitivity test
DZN: Diazinon
GPx: Glutathione peroxidase
NF-κB: Nuclear factor-κB
Ops: Organophosphorus pesticides
ROS: Reactive oxygen species
SOD: Superoxide dismutase
SRBCs: Sheep red blood cells
TNF-α: Tumour necrosis factor-alpha
